# Feeding and metabolic consequences of scheduled consumption of large, binge-type meals of high fat diet in the Sprague–Dawley rat

**DOI:** 10.1016/j.physbeh.2014.01.018

**Published:** 2014-04-10

**Authors:** T. Bake, D.G.A. Morgan, J.G. Mercer

**Affiliations:** a University of Aberdeen, Rowett Institute of Nutrition and Health, Ingestive Behaviour Group, Bucksburn, Aberdeen, UK; b AstraZeneca, Mereside, Alderley Park, Macclesfield, UK

**Keywords:** Temporal feeding analysis, Palatability, Scheduled feeding, Binge eating, Diet-induced obesity, Rat

## Abstract

Providing rats and mice with access to palatable high fat diets for a short period each day induces the consumption of substantial binge-like meals. Temporal food intake structure (assessed using the TSE PhenoMaster/LabMaster system) and metabolic outcomes (oral glucose tolerance tests [oGTTs], and dark phase glucose and insulin profiles) were examined in Sprague–Dawley rats given access to 60% high fat diet on one of 3 different feeding regimes: *ad libitum* access (HF), daily 2 h-scheduled access from 6 to 8 h into the dark phase (2 h-HF), and twice daily 1 h-scheduled access from both 1–2 h and 10–11 h into the dark phase (2 × 1 h-HF). Control diet remained available during the scheduled access period. HF rats had the highest caloric intake, body weight gain, body fat mass and plasma insulin. Both schedule-fed groups rapidly adapted their feeding behaviour to scheduled access, showing large meal/bingeing behaviour with 44% or 53% of daily calories consumed from high fat diet during the 2 h or 2 × 1 h scheduled feed(s), respectively. Both schedule-fed groups had an intermediate caloric intake and body fat mass compared to HF and control (CON) groups. Temporal analysis of food intake indicated that schedule-fed rats consumed large binge-type high fat meals without a habitual decrease in preceding intake on control diet, suggesting that a relative hypocaloric state was not responsible or required for driving the binge episode, and substantiating previous indications that binge eating may not be driven by hypothalamic energy balance neuropeptides. In an oGTT, both schedule-fed groups had impaired glucose tolerance with higher glucose and insulin area under the curve, similar to the response in *ad libitum* HF fed rats, suggesting that palatable feeding schedules represent a potential metabolic threat. Scheduled feeding on high fat diet produces similar metabolic phenotypes to mandatory (no choice) high fat feeding and may be a more realistic platform for mechanistic study of diet-induced obesity.

## Highlights

• Rapid adaptation of feeding behaviour to scheduled palatable diet access
• No evidence of reduced feeding (hypophagia) prior to scheduled palatable meals
• Schedule-fed rats exhibit adverse metabolic symptoms.

## 1 Introduction

Genetic rodent models of obesity caused by single-gene mutations, and engineered transgenic lines, have contributed substantially to understanding of the control of energy intake and body weight [1–3]. Other investigations of energy homeostasis have been conducted using rodent models of diet-induced obesity (DIO) through *ad libitum* but usually obligatory feeding of high fat or high energy diets [4,5]. However, it can be questioned whether such dietary manipulations are the most appropriate to model human eating behaviour leading to obesity [5], where the meal is a key element in food intake and eating behaviour [6]. In this regard, a rodent model of scheduled access to palatable diet without food restriction may be more appropriate to mimic human eating behaviour and the development of overweight and obesity.

One such scheduled feeding regime was reported by Berner et al. in 2008 [7] to induce substantial food intake over short periods of time in rats [7]. Utilising this model, we provided scheduled access to a solid high fat palatable diet for a 2 h-period each day without imposed caloric restriction during the remainder of the day, a manipulation that resulted in consumption of large binge-type meals in both rats and mice [8]. Despite the size of these feeding events and their relatively short duration, our previous examination of this model in the two species did not provide evidence of any potentially causative perturbation in expression of hypothalamic homeostatic neuropeptide genes that might be driving consumption [8]. This finding suggested that the schedule-fed animals were not in negative energy balance in advance of the initiation of the large meals, but also served to highlight that the effect of such dietary regimes on the temporal structure of feeding and on other aspects of behaviour and metabolism is largely unknown. Consequently, we have undertaken further characterisation of this large meal/binge eating model at a behavioural and metabolic level, focussing on how schedule feeding the palatable diet changes the temporal food intake pattern on the control diet, the metabolic consequences of imposing this regime, and an evaluation of pre-meal gut and metabolic hormones. In addition, we extend the model beyond a single large meal of palatable diet to assess the outcome of a feeding regime of two 1 h access periods per day.

## 2 Methods and materials

### 2.1 Animals

32 male Sprague–Dawley rats with an initial body weight of 190–200 g (Charles River Laboratories, Margate, UK) were acclimatized in groups in a reversed 12 h:12 h light–dark cycle (lights off at 09:00, ZT12; lights on at 21:00, ZT0; ZT, zeitgeber time). After two weeks, rats were single housed in TSE PhenoMaster/LabMaster feeding/drinking cages (TSE Systems, Bad Homburg, Germany) and acclimatized for a further week before the start of a week of baseline food intake measurements. All animals had *ad libitum* access to a standard pellet diet (Special Diets Services, Witham, UK, #871505 CRM (P); 22% protein, 69% carbohydrate, 9% fat by energy, 2.67 kcal/g) unless otherwise stated. Water was freely available at all times. All procedures were licenced under the Animals (Scientific Procedures) Act of 1986 and approved by the CVGI ethical committee of AstraZeneca, Alderley Park.

### 2.2 Dietary manipulation

Following acclimatization, animals were allocated to one of four weight-matched and baseline food-intake-matched groups (body weight 407.9 ± 8.6 g, food intake 86.2 ± 2.6 kcal, n = 8) with the following feeding regimes: (1) *ad libitum* access to standard pellet diet for 24 h per day (CON), (2) *ad libitum* access to high fat diet (Research Diets, New Brunswick, NJ, USA, D12492; 20% protein, 20% carbohydrate, 60% fat by energy, 5.24 kcal/g) for 24 h per day (HF), (3) *ad libitum* access to standard pellet diet for 24 h per day and scheduled access to high fat diet for 2 h per day during the mid-dark phase from ZT18 to ZT20 (2 h-HF), and (4) *ad libitum* access to standard pellet diet for 24 h per day and scheduled access to high fat diet for 1 h twice per day during early and late dark phase from ZT13 to ZT14 and from ZT22 to ZT23 (2 × 1 h-HF). The timing of the scheduled feeding was chosen for the 2 h-HF group to precisely replicate the feeding paradigm described by Berner et al. [7] and Bake et al. [8], and for the 2 × 1 h-group to effectively divide the intake into two substantial meals within the dark phase. The dietary manipulation continued for 6 weeks during which food intake temporal structure, glucose and insulin profiles and oral glucose tolerance tests (oGTTs) were examined. Animals were then killed by CO_2_ inhalation in the middle of the dark phase (at intervals from ZT17) for groups CON, HF and 2 h-HF or at the beginning of the dark phase (at intervals from ZT12) for the 2 × 1 h-HF group. Termination time of 1 h prior to scheduled presentation of the high fat diet was chosen so that blood samples would reflect the pre-meal response of anticipatory hormones and metabolites in the schedule-fed groups. Terminal blood samples were collected by cardiac puncture. Body composition was determined postmortem using a MRI system (Echo Medical Systems, Houston, TX, USA).

### 2.3 Food intake measurement and temporal food intake analysis

In the last week of the acclimatization period (baseline week) and over the 6 weeks of the dietary manipulation, food intake was measured using the TSE PhenoMaster/LabMaster system, which automatically records the weight of food eaten. Each cage had two sets of calibrated food sensors that separately recorded food eaten to a sensitivity of 0.01 g, one of which had an automatically-controlled sleeve permitting access to the high fat diet only during scheduled feeding. Food spillage was minimised by a catch tray. Cumulative food intake was recorded at intervals of 15 min, and summarised in 1 h intervals and then averaged per animal and study week for statistical analysis.

### 2.4 Glucose profiles, insulin profiles and oral glucose tolerance tests

Glucose and insulin profiles were analysed on 3 occasions, at baseline, and after 2 or 5 weeks on the respective feeding regimes. Blood samples were taken at 8 time points across the scheduled feeding period of the 2 h-HF group and the first scheduled feeding period of the 2 × 1 h-HF group: 1 h before the start of the dark phase (ZT11), at the start of the dark phase (ZT12) and over the course of the dark phase (at ZT13, ZT14, ZT16, ZT18 and ZT20). In addition, oral glucose tolerance tests (oGTTs) were performed after 4 weeks on the feeding regime. Rats were fasted for 22 h and then received an oral glucose dose of 3 g/kg by gavage in the middle of the dark phase at ZT18. Blood samples were taken before the oral glucose dose (0 min) and then at the following time points: 20 min, 40 min, 60 min, 90 min and 120 min. Blood samples for both the profiles and the oGTTs were drawn by tail prick and immediately measured for glucose (Accu-Chek Aviva; Roche Diagnostics Ltd., Burgess Hill, UK). For insulin measurement, a 20 μl blood sample was taken into a heparinised capillary tube and plasma insulin concentrations were measured using a rat-specific ELISA kit (#90060; Crystal Chem Inc., Downers Grove, IL, USA). The sensitivity of the assay was 0.1 ng/ml, and the intra-assay coefficient of variation (CV) was 7.65%. Area under the curve (AUC) for both glucose and insulin oGTT was calculated with the trapezoidal rule using concentrations corrected for baseline.

### 2.5 Circulating hormones and metabolites

Terminal blood samples were assayed for leptin, insulin, ghrelin, GLP-1, glucose, triglycerides and non-esterified fatty acids (NEFA). Serum leptin concentrations were measured using a rat-specific radioimmunoassay kit (#RL-83K; LINCO Research, Poole, UK). The sensitivity was 0.5 ng/ml and the intra-assay CV was 5.37%. Plasma insulin was measured as above. Total ghrelin concentrations in plasma were measured using a rat/mouse specific multi-array assay kit (#K150IOC-1; Meso Scale Discovery, Gaithersburg, MD, USA) with a sensitivity of 1.3 pg/ml and an intra-assay CV of 3.94%. Active GLP-1 concentrations in plasma were determined using a multi-species ELISA kit (#EGLP-35K; Millipore, Billerica, MA, USA). The sensitivity of the assay was 2 pg/ml and the intra-assay CV was 6.09%. Blood for active GLP-1 assay was collected into EDTA-plasma tubes containing 10 μl DPP-IV inhibitor (#DPP4; Millipore, Billerica, MA, USA) and 500 Ki unit aprotinin protease inhibitor (#10981532001; Roche Diagnostics Ltd., Burgess Hill, UK) per 1 ml blood. Plasma glucose, triglycerides and NEFA were determined using the fully automated Konelab analyser (Thermo Fisher Scientific, Waltham, MA, USA). The sensitivities of the assays were 0.3 mmol/l, 0.05 mmol/l and 0.01 mmol/l, respectively, with intra-assay CVs of 1.32%, 1.08% and 1.67%, respectively.

### 2.7 Statistical analysis

Statistical analysis employed SigmaPlot 11.0 software (Systat Software, Chicago, IL, USA). To reveal effects of dietary manipulation between the 4 groups, data were analysed by one-way analysis of variance (ANOVA) or by Kruskal–Wallis one-way ANOVA on ranks when the data were not normally distributed and/or variances were not equal. Repeated measurements for food intake, oGTT, glucose profile or insulin profile data were analysed with two-way repeated measures (RM) ANOVA for effect of diet and time point and for interactions. Post hoc and planned comparisons were assessed with Student–Newman–Keul (SNK) tests. Correlations between variables were analysed by Spearman Rank Order Correlation. Outcomes were considered statistically significant if P values were less than 0.05. The data are presented as group mean ± SEM.

## 3 Results

### 3.1 Food intake

**Fig. 1:**
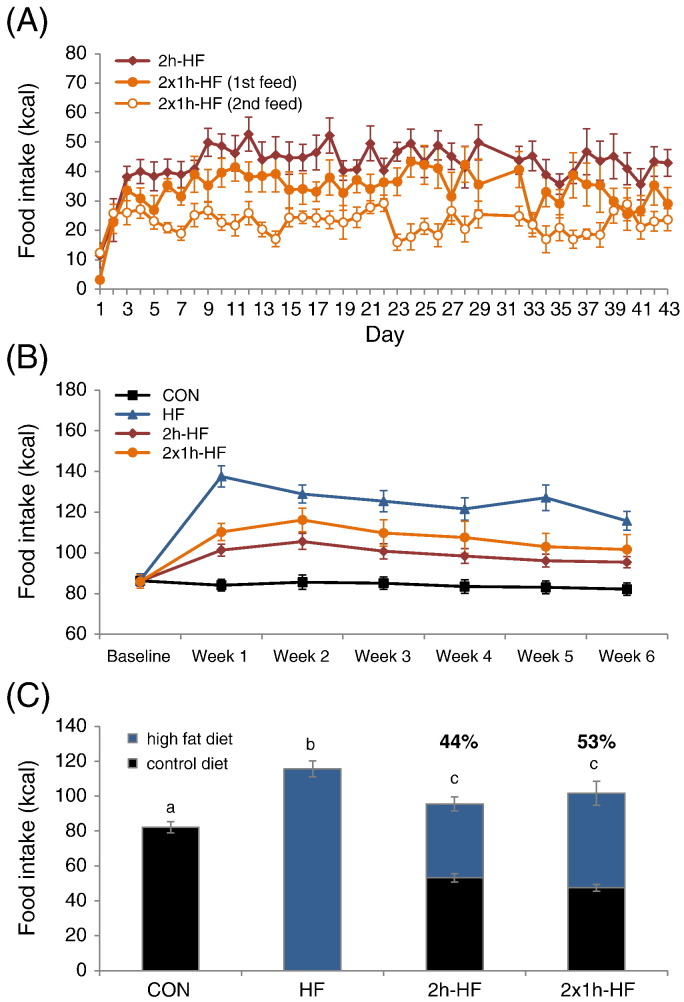
Caloric intake of Sprague–Dawley rats fed on a high fat diet with *ad libitum* or scheduled access. (A) Caloric intake during scheduled feeding time for both schedule-fed groups over 6 weeks. (B) Total daily caloric intake during baseline and each week of the dietary manipulation. (C) Total daily caloric intake during week 6 of the dietary manipulation showing calories consumed from control diet and high fat diet. Percentages above bars refer to calories consumed from high fat diet during scheduled feeding time by schedule-fed rats relative to total 24 h intake. Grey bars, calories derived from control diet; black bars, calories derived from high fat diet. Different letters indicate *P* < 0.05 by one-way ANOVA and Student–Newman–Keul post hoc test for total caloric intake. Data are presented as mean ± SEM.

Both schedule-fed groups rapidly adapted their feeding behaviour to scheduled access conditions (Fig. 1A). Maximum intakes were reached during the second week of dietary manipulation. In general, more high fat diet was consumed during the first 1 h-access period than during the second 1 h-access period in the 2 × 1 h-HF group (two-way RM ANOVA; P = 0.037), but consumption during either period tended to be lower (first access, P = 0.108) or was routinely lower (second access, P < 0.001) than that of the 2 h-HF group during their 2 h access, although cumulative high fat diet intake was higher in the 2 × 1 h-HF group (P = 0.018).

Total daily caloric intake prior to and during weeks 1 to 6 of the dietary manipulation showed a significant interaction between diet group and time point (Fig. 1B) (two-way RM ANOVA; P < 0.001). Intake was not different between the groups during the baseline week prior to dietary manipulation. During weeks 1 to 6 on dietary manipulation, total daily caloric intake differed between the groups: rats fed *ad libitum* on high fat diet had the highest caloric intakes (SNK; P < 0.05 vs. CON, 2 h-HF and 2 × 1 h-HF) and schedule-fed rat groups had intermediate caloric intakes (SNK; P < 0.05 vs. CON and HF) during all weeks of the dietary manipulation, with the exception that *ad libitum* fed HF rats showed only a trend towards a higher caloric intake compared to 2 × 1 h-schedule-fed rats during week 2 (SNK; P = 0.054). Over the course of the study, *ad libitum* HF rats and both schedule-fed groups had elevated caloric intake in weeks 1 to 6 vs. baseline week (SNK; P < 0.001). *Ad libitum* HF rats had the highest caloric intake in week 1 vs. weeks 2 to 6 (SNK; P < 0.001), whereas schedule-fed rats had the highest caloric intake in week 2 (SNK; P < 0.05 vs. weeks 4, 5, and 6 for 2 h-HF and vs. all other weeks for 2 × 1 h-HF).

Schedule-fed rats showed large meal/bingeing behaviour such that on week 6, 44.4% of daily calories were consumed from high fat diet during the 2 h-schedule feed (38.4% during first hour and 6.0% during the second hour), whereas for the 2 × 1 h-HF group the equivalent figure was 53.3% when the two 1 h-scheduled feeds were combined (31.5% and 21.8%, respectively; Fig. 1C). High fat diet supplied either in a 2 h-access period or in 2 × 1 h periods displaced calories consumed from control diet from total daily intake, but compensation was incomplete.

### 3.2 Temporal food intake analysis

**Fig. 2:**
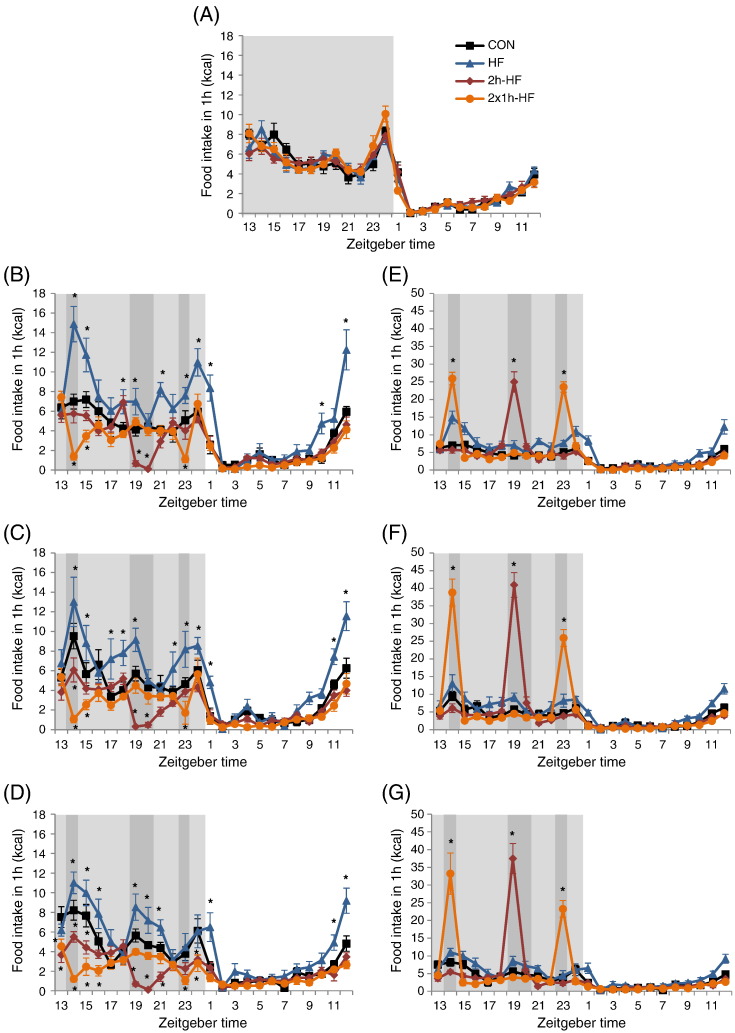
Temporal food intake analysis during baseline week (A), and week 1 (B, E), week 2 (C, F) and week 6 (D, G) of dietary manipulation. (B–D) Temporal food intake structure showing caloric intake excluding that from high fat diet during scheduled feeding. (E–G) Temporal food intake structure showing total caloric intake including calories from high fat diet during scheduled feeding. Light shaded area indicates dark phase; dark shaded area indicates scheduled access to high fat diet. *P < 0.05 vs. CON by two-way repeated measures ANOVA and Student–Newman–Keul post hoc test. For clarity, *P* < 0.01 and *P* < 0.001 are not differentiated from *P* < 0.05, and diagrams (E–G) display only significant differences during scheduled feeding time for 2 h-HF and 2 × 1 h-HF vs. CON (ZT14, ZT19, ZT20 and ZT23). Data are presented as mean ± SEM.

Temporal food intake structure was analysed during the baseline week and each subsequent week. Since total daily caloric intake showed differences between baseline, week 1 and week 2, but not amongst weeks 3 to 6 (Fig. 1B), only data for baseline, week 1, week 2 and week 6 are shown in Fig. 2. During baseline, temporal structure did not differ between the groups, which were therefore pooled for analysis. There was an effect of time on caloric intake (two-way RM ANOVA; P < 0.001; Fig. 2A). Statistical analysis supported the existence of three peaks in intake in the baseline week. Caloric intake started to increase at the end of the light phase (SNK; at ZT9 to ZT12 (e.g. data from ZT11 to ZT12), P < 0.05 vs. all other ZT intervals), peaking at the beginning of the dark phase (SNK; at ZT13 to ZT15, P < 0.05 vs. all other ZT intervals). A second, lower, peak occurred at the mid of the dark phase (SNK; at ZT20, P < 0.05 vs. all other ZT intervals), and the third and highest peak occurred during the last hour of the dark phase (SNK; at ZT24, P < 0.05 vs. all other ZT intervals).

During each week of the dietary manipulation there was a significant interaction between time and diet (two-way RM ANOVA; P < 0.001) for caloric intake excluding calories from scheduled feeding (Fig. 2B–D for weeks 1, 2 and 6) as well as for caloric intake including calories from scheduled feeding (Fig. 2E–G). For simplicity, only comparisons with the control group (CON) are discussed and shown on Fig. 2. For *ad libitum* HF fed rats, caloric intake on high fat diet was increased during the last hour of the light phase at ZT12 (P < 0.001 vs. CON) and also during at least half of the 1 h intervals over the dark phase. For example, for week 6 this applied to ZT14 to ZT16 (P = 0.004, P = 0.018, P = 0.004 vs. CON), ZT19 to ZT21 (P = 0.003, P = 0.010, P = 0.034 vs. CON), and during the first hour of the light phase (ZT1, P < 0.001 vs. CON). Caloric intake of 2 h-HF schedule fed rats was not different from CON rats during the whole light phase. By contrast, caloric intake from control diet was decreased at the start of the dark phase, from ZT13 to ZT15; this effect was most apparent at week 6 (ZT13, P < 0.001; ZT14, P = 0.006; ZT15, P < 0.001 vs. CON for week 6), but no differences in caloric intake occurred during the 3 h running up to scheduled feeding (ZT16 to ZT18). Caloric intake from control diet was decreased to less than 1 kcal during the scheduled feeding period (ZT19 and ZT20, P < 0.001 vs. CON for week 6), whereas caloric intake from high fat diet was increased during the first hour of scheduled feeding only (ZT19, P < 0.001 vs. CON for weeks 1 to 6). Caloric intake from control diet was further decreased during the hour after scheduled feeding (ZT21, P = 0.008 vs. CON for week 6) and during the last hour of the dark phase (ZT24, P = 0.008 vs. CON for week 6). 2 × 1 h-HF schedule fed rats also had a caloric intake that did not differ from CON rats during the whole light phase. Caloric intake from control diet was decreased during the hour before the first schedule feed (ZT13), but only at week 6 (P = 0.007 vs. CON), during both scheduled feeds (ZT14, P < 0.001; ZT23, P = 0.015 vs. CON for week 6) and after the schedule feeds (ZT15, P < 0.001; ZT16, P = 0.006; ZT24, P = 0.004 vs. CON for week 6). There was no reduction in intake of control diet before the second scheduled feed. Caloric intake from high fat diet was increased during both 1 h scheduled feeds (ZT14 and ZT23, P < 0.001 vs. CON for weeks 1 to 6).

**Fig. 3:**
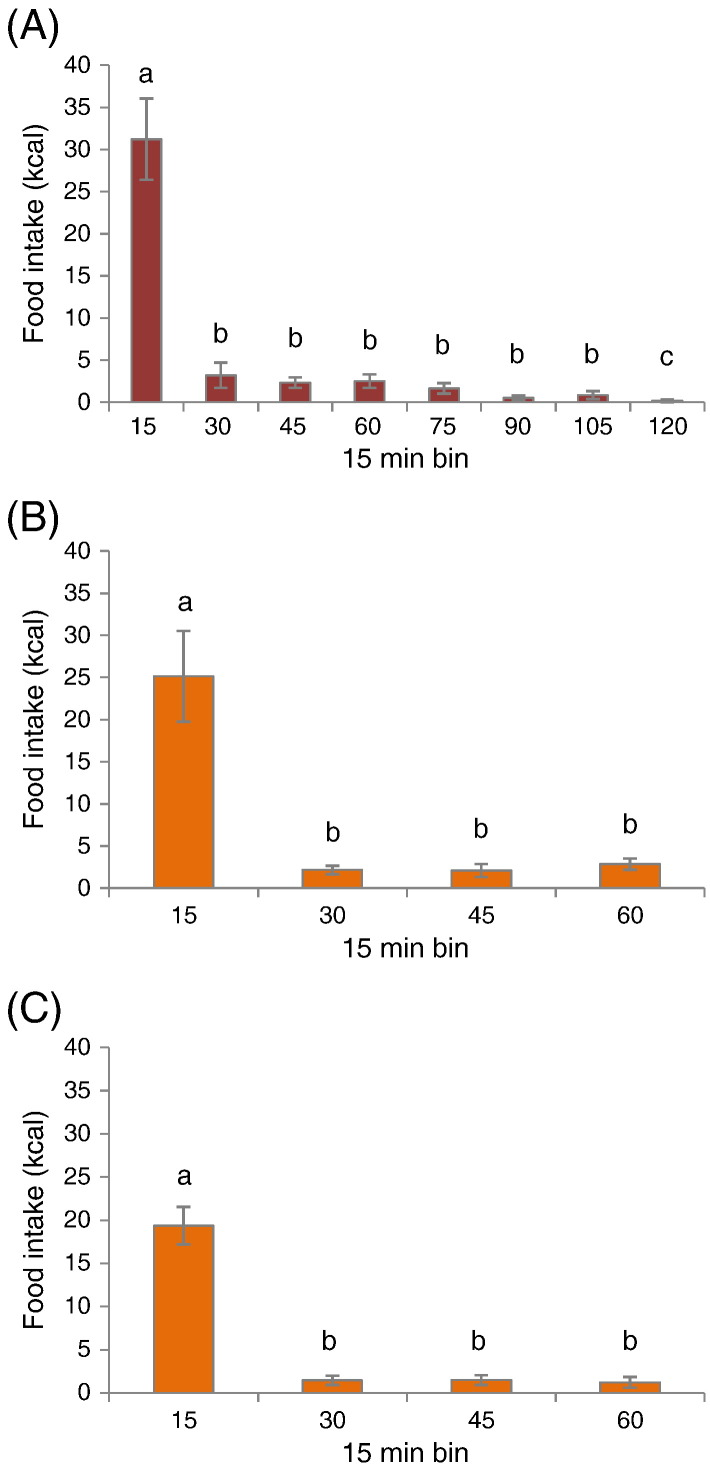
Temporal analysis of high fat diet intake in 15 min bins during scheduled feeding in week 6 of dietary manipulation. Caloric intake from high fat diet during (A) the 2 h-access in 2 h-HF rats, and (B) the first 1 h-access and (C) the second access in 2 × 1 h-HF rats. Data are shown as absolute values; mean ± SEM. Different letters indicate *P* < 0.05 by one-way ANOVA and Student–Newman–Keul post hoc test.

Further temporal analysis of the food intake in 15 min bins during the schedule-fed periods revealed that both schedule-fed groups consumed most of their calories during the first 15 min following presentation (Fig. 3A–C). For the 2 h-HF group this represented 73.5% of 2 h intake (one-way ANOVA; P < 0.05 vs. all other bins), whereas for 2 × 1 h-HF rats this was 78.0% and 82.3% of their respective 1 h caloric intake during the first or second access period (one-way ANOVA; P < 0.05 vs. all other bins).

### 3.3 Body weight and composition

**Fig. 4:**
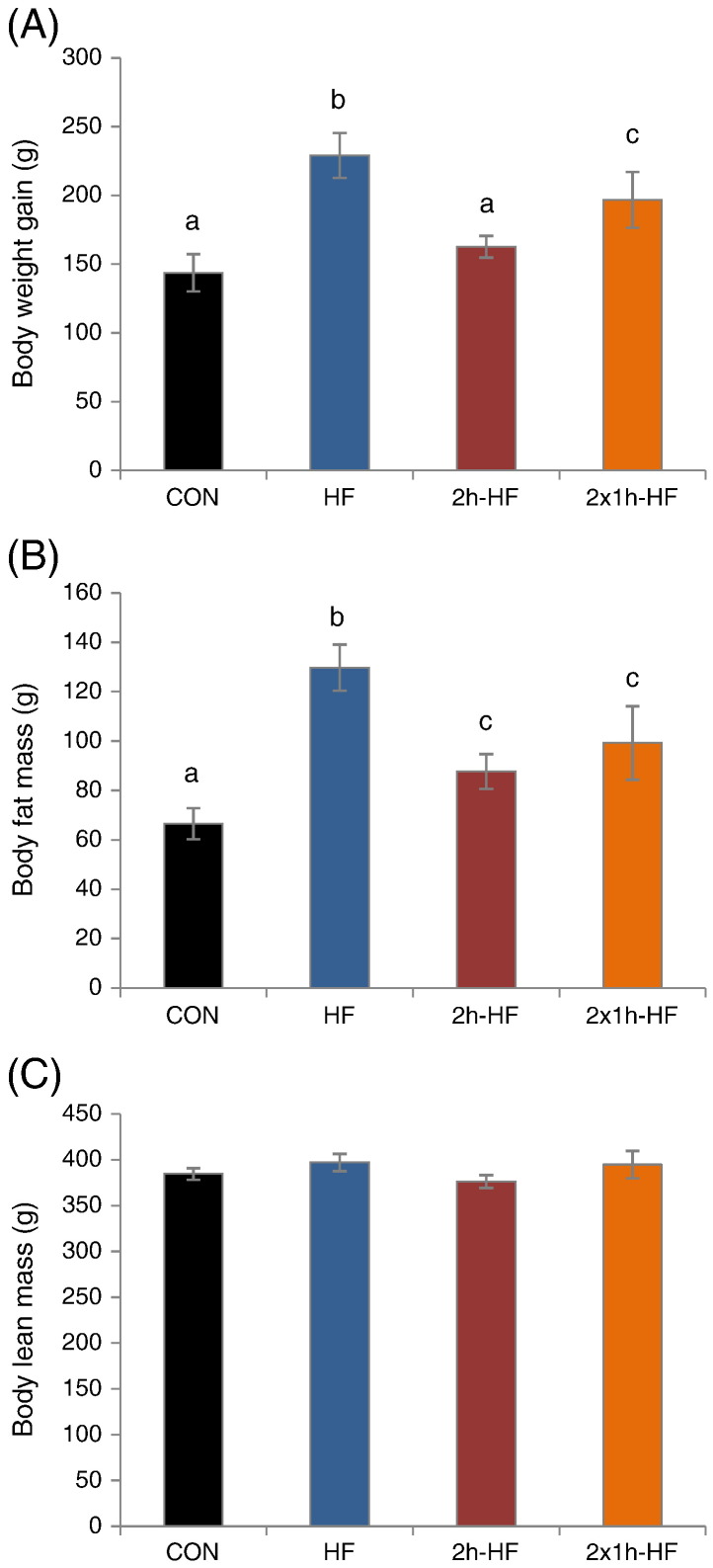
Body composition parameters in Sprague–Dawley rats fed on a high fat diet with *ad libitum* or scheduled access for 6 weeks: (A) Body weight gain, (B) body fat mass and (C) body lean mass. Data are presented as mean ± SEM. Different letters indicate *P* < 0.05 by one-way ANOVA and Student–Newman–Keul post hoc test.

The dietary manipulation had effects on body weight gain and body fat mass (Fig. 4A and B) (one-way ANOVA; P = 0.002, P < 0.001, respectively), but not body lean mass (Fig. 4C). *Ad libitum* HF rats had the highest body weight gain and body fat mass (SNK; P < 0.05 vs. CON, 2 h-HF and 2 × 1 h-HF), whereas 2 × 1 h-HF-schedule-fed rats had intermediate values (SNK; P < 0.05 vs. CON and HF). 2 h-Schedule-fed rats had also an intermediate body fat mass (SNK; P < 0.05 vs. CON and HF), whereas body weight gain did not differ from CON.

### 3.4 Metabolic profiles

**Fig. 5:**
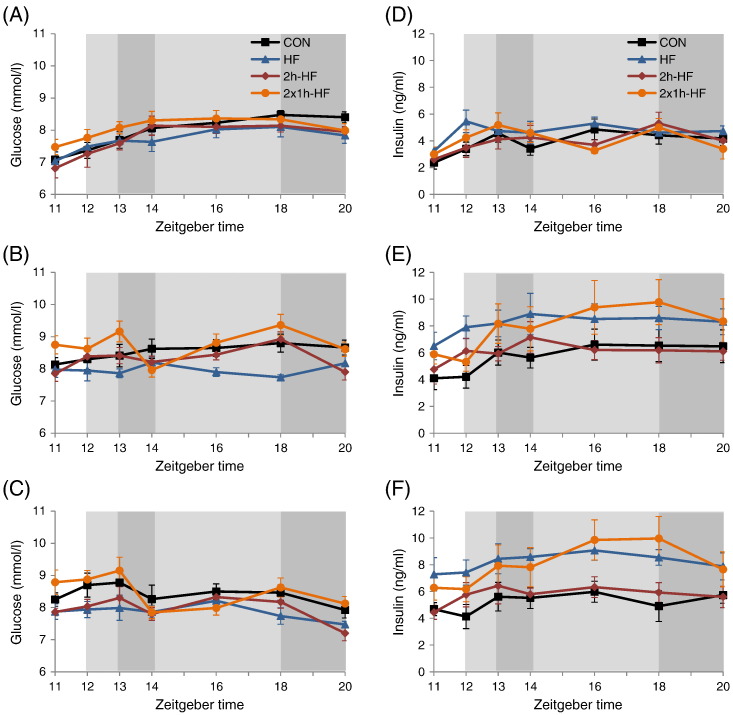
Blood glucose and plasma insulin profiles in Sprague–Dawley rats fed on a high fat diet with *ad libitum* or scheduled access. Blood glucose and plasma insulin profiles were analysed at 3 longitudinal time points: in the last week of the acclimatization phase (A, D), after 2 weeks on the feeding paradigm (B, E) and after 5 weeks on the feeding paradigm (C, F). Blood samples were taken before and over the course of the dark phase while the rats were fed on their respective diets. Light shaded area indicates dark phase; dark shaded area indicates scheduled access to high fat diet. Data are presented as mean ± SEM.

Blood glucose profiles were measured at baseline, and after 2 or 5 weeks on dietary manipulation. At baseline, there was an overall effect of time on glucose profile (Fig. 5A) (two-way RM ANOVA; P < 0.001). Glucose concentrations were lower at ZT11 (1 h before the dark phase) compared to all dark phase time points (SNK, P = 0.007, P < 0.001, P < 0.001, P < 0.001, P < 0.001, P < 0.001, respectively), and at ZT12 (start of the dark phase) compared to ZT14, ZT16, ZT18 and ZT20 (SNK; P = 0.008, P < 0.001, P < 0.001 and P = 0.005, respectively). After 2 weeks on the respective feeding regimes, there was a significant interaction between diet and time (Fig. 5B) (two-way RM ANOVA; P = 0.002), with evidence of elevated blood glucose in schedule-fed groups at the onset of access to high fat diet. In the 2 h-HF schedule-fed group, glucose concentration peaked at ZT18 (SNK; P = 0.003, P = 0.089, P = 0.086 and P = 0.004; ZT18 vs. ZT11, ZT14, ZT16 and ZT18; P = 0.003 vs. HF), whereas a peak in the 2 × 1 h-HF schedule-fed group occurred at ZT13, the start of the first scheduled access period (SNK; P < 0.001; ZT13 vs. ZT14; P = 0.036, P = 0.002 and P = 0.089 vs. CON, HF and 2 h-HF). In addition, glucose concentrations at ZT14 were decreased compared to all other time points (SNK; P = 0.028, P = 0.050, P < 0.001, P = 0.023, P < 0.006 and P = 0.022 vs. ZT11, ZT12, ZT13, ZT16, ZT18 and ZT20). Glucose concentrations in *ad libitum* HF rats were lower than CON in the mid-dark phase (SNK; P = 0.089 and P = 0.003 vs. CON at ZT16 and ZT18). Similar patterns were observed after 5 weeks on the respective feeding regimes; there was an overall effect of time (Fig. 5C) (two-way RM ANOVA; P < 0.001). The 2 h-HF schedule-fed group again exhibited a reduction in glucose concentration across the scheduled feeding period (ZT18 to ZT20; SNK; P = 0.058, P = 0.020, P = 0.002, P = 0.038, P = 0.002 and P = 0.007; ZT20 vs. ZT11, ZT12, ZT13, ZT14, ZT16 and ZT18). Glucose concentrations in the 2 × 1 h-HF scheduled fed group were again decreased at ZT14 (SNK; P = 0.011, P = 0.005, P < 0.001 and P = 0.037; ZT14 vs. ZT11, ZT12, ZT13 and ZT18).

There were significant effects of time on insulin profile at baseline, and after 2 and 5 weeks (Fig. 5D–F) (two-way RM ANOVA; P < 0.001). At baseline, insulin concentrations were lower at ZT11 compared to all other time points (SNK; P < 0.001), whereas during dietary manipulation, insulin concentrations were lower at ZT11 and ZT12 compared to the other time points (SNK; P < 0.001 and P < 0.05 after 2 weeks; P < 0.05 and P < 0.05 after 5 weeks). In addition, after 5 weeks on dietary manipulation, plasma insulin profiles also showed an effect of diet (Fig. 5F) (two-way RM ANOVA; P = 0.042), which had begun to emerge at 2 weeks (Fig. 5E), whereby *ad libitum* HF rats and 2 × 1 h-HF schedule-fed rats had elevated insulin concentrations compared to CON and 2 h-HF schedule fed rats: *ad libitum* HF rats showed a trend to increased insulin at ZT14 (P = 0.092 vs. CON; P = 0.079 vs. 2 h-HF), ZT16 (P = 0.090 vs. CON; P = 0.062 vs. 2 h-HF) and ZT18 (P = 0.091 vs. CON), whereas 2 × 1 h-HF schedule-fed rats had increased insulin at ZT16 (P = 0.045 vs. CON and 2 h-HF) and ZT18 (P = 0.018 vs. CON; P = 0.055 vs. 2 h-HF).

### 3.5 Oral glucose tolerance tests

**Fig. 6:**
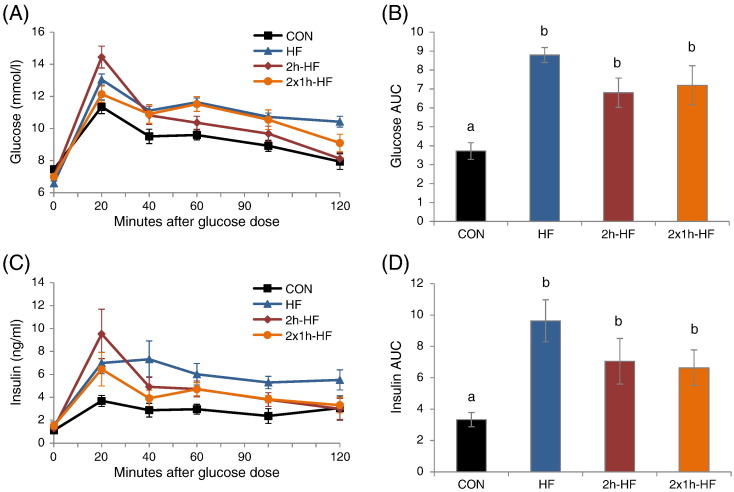
Response to an oral glucose tolerance test (oGTT) in Sprague–Dawley rats after 4 weeks on dietary manipulation. (A) Blood glucose curves. (B) Areas under curve (AUC) for glucose response from 0 to 120 min. (C) Plasma insulin curves. (D) AUC for insulin response from 0 to 120 min. AUC calculated from concentrations corrected for baseline. Different letters indicate *P* < 0.05 by one-way ANOVA and Student–Newman–Keul post hoc test. Data are presented as mean ± SEM.

oGTTs were performed after 4 weeks on the dietary feeding regime. Fasting glucose was significantly different between the groups (7.4 ± 0.2 mmol/l, 6.6 ± 0.1 mmol/l, 7.0 ± 0.1 mmol/l and 7.0 ± 0.2 mmol/l for CON, HF, 2 h-HF and 2 × 1 h-HF rats) (one-way ANOVA; P = 0.008), with decreased glucose concentration in *ad libitum* HF rats, 2 h-HF rats and 2 × 1 h-HF rats (SNK; P = 0.004, P = 0.055 and P = 0.094 vs. CON). For blood glucose concentration over the course of the oGTT, there was a significant interaction between diet and time (Fig. 6A) (two-way RM ANOVA; P < 0.001). In general, all glucose curves showed a peak at 20 min and there was a later decline in CON and schedule-fed rats that was blunted in *ad libitum* HF rats. The glucose AUC was also affected by the dietary manipulation (Fig. 6B; one-way ANOVA; P < 0.001), being higher in *ad libitum* HF fed, 2 h-schedule-fed and 2 × 1 h-schedule-fed rat groups (SNK; P < 0.001, P = 0.005, P = 0.005 vs. CON).

Fasting insulin did not differ between the groups (1.1 ± 0.2 ng/ml, 1.6 ± 0.3 ng/ml, 1.3 ± 0.2 ng/ml, 1.5 ± 0.3 ng/ml for CON, HF, 2 h-HF and 2 × 1 h-HF rats). Insulin concentrations in response to the oGTT showed an interaction between diet group and time (Fig. 6C) (two-way RM ANOVA; P = 0.044). Insulin concentrations did not differ between time points in control rats. Schedule-fed rats showed a peak in insulin concentration at 20 min and a later decline. *Ad libitum* HF rats had increased insulin concentrations across the 120 min test. The insulin AUC was also affected by the dietary manipulation (Fig. 6D) (one-way ANOVA; P = 0.004), being higher in *ad libitum* HF fed, 2 h-schedule-fed and 2 × 1 h-schedule-fed rat groups (SNK; P < 0.05 vs. CON).

### 3.6 Circulating hormones and metabolites

The hormones, leptin, insulin, ghrelin and GLP-1, and the metabolites, glucose, triglyceride and NEFA were measured in the terminal blood sample (Table 1). One-way ANOVA revealed significant effects on leptin, insulin, triglyceride and NEFA concentrations (P < 0.001, P = 0.003, P = 0.003 and P < 0.001, respectively). *Ad libitum* HF rats had the highest concentrations of leptin (SNK; P < 0.001 vs. CON, 2 h-HF and 2 × 1 h-HF), insulin (SNK; P = 0.013, P = 0.004 and P = 0.007 vs. CON, 2 h-HF and 2 × 1 h-HF) and NEFA (SNK; P < 0.001 vs. CON, 2 h-HF and 2 × 1 h-HF), whereas concentrations in schedule-fed groups did not differ from controls. Triglyceride concentrations were lower in all three HF-fed groups compared to controls (SNK; P = 0.006, P = 0.004 and P = 0.011, respectively). Ghrelin, GLP-1 and glucose concentrations did not differ between the groups.

## 4 Discussion

Providing rats with a palatable high fat diet for a 2 h-period each day without caloric restriction, in this case in addition to continued *ad libitum* access to control diet, is very effective in promoting hyperphagia for the time that the palatable diet is available, increasing body fat mass and body weight gain, depending on the scheduled feeding regime used. Provision of two 1 h meals marginally increased caloric intake and HF diet intake leading to higher weight gain compared to a single 2 h meal, although accompanying increases in post-mortem body fat were not statistically significant. Consistent with previous reports when control diet was replaced during schedule feeding [7,8], rats rapidly adapted their feeding behaviour to scheduled access conditions and binged on the palatable high fat diet when it was offered (Fig. 1). Rats fed *ad libitum* on the same high fat diet acted as positive controls with development of DIO, elevated leptin levels and increased body weight gain.

Whereas our previous study [8] found no evidence of potentially causative perturbation in expression of hypothalamic homeostatic neuropeptide genes, such as neuropeptide Y or cocaine- and amphetamine-regulated transcript, that might contribute to the consumption of such a large binge-type meal, information on temporal energy intake patterns was limited to manual weigh-back of food consumed in the 2 h scheduled feeding period and the remaining 22 h period. More detailed information on the temporal structure of food intake prior to the scheduled feeding period and the impact of scheduled feeding on blood hormones and metabolites will provide important context to the gene expression levels observed in these animals [8]. Consequently, in this follow-up study we focused on: (i) differences in the temporal structure of feeding behaviour between schedule-fed rats (2 h-HF or 2 × 1 h-HF) and control (CON or HF) rats, (ii) the metabolic consequences of high fat feeding under these regimes, and (iii) hormones that might be involved in the anticipation of the scheduled meals.

During baseline *ad libitum* feeding conditions, rats displayed a clear diurnal rhythm of food intake, consuming most of their food during the active dark phase, with clear peaks at the beginning and the end of the dark phase (Fig. 2A) [9,10]. *Ad libitum* high fat feeding was characterised by amplified caloric intake peaks at the beginning, middle, and end of the dark phase (Fig. 2B–D). Larger feeding bout sizes have been reported previously in *ad libitum* fed rats on high fat pellet [11] or high fat liquid diets [12], as well as in rats prone to DIO compared to DIO resistant rats fed on a high fat diet [13]. A*d libitum* intake in HF rats was maximal during week 1, likely reflecting a combination of novelty and increased palatability compared to the control diet [11].

When rats were schedule-fed and, as a consequence, food consumption was displaced to times when the palatable high fat diet was available, the food intake rhythm observed during baseline persisted albeit with a lower amplitude during the remainder of the day. There were no differences in light phase food intake between schedule-fed and CON rats, and no major shift in feeding episodes on control diet towards different time points during the dark phase. Crucially, schedule-fed rats did not routinely decrease their caloric intake below that of CON rats within the 2–3 h period prior to scheduled feeding, with the exception of 2 × 1 h-HF rats in week 6, which had a lower intake of control diet during the hour before the first access, although this may reflect CON rats having an elevated intake during this one hour period on week 6. Overall, these observations indicate that schedule-fed rats were not in a hypocaloric, negative energy balance, state prior to schedule feeding, in accord with our previous observations that large meals of palatable food do not appear to be driven by homeostatic neuropeptides in the arcuate nucleus of the hypothalamus [8]. The broadly unchanged food intake and hypothalamic gene expression prior to scheduled meals is noteworthy since increased food anticipatory activity (FAA) – arousal, locomotor activity and body core temperature – has been reported within this time frame, both in restricted feeding schedules when rats are trained to eat their entire daily food during a short time [14–16], and in palatable feeding schedules when rats receive timed access to a palatable food in addition to *ad libitum* control diet [14,15,17]. However, in some studies with palatable feeding schedules, FAA occurred with a lower intensity [15], or not in all rats of the study population [14].

Temporal analysis of food intake in 15-min bins suggested that a state approaching satiety was reached during the first 15 min of each scheduled feeding period (Fig. 3A–C). Although during baseline all rats had their highest overall intake within the last hour of the dark phase, 2 × 1h-HF schedule-fed rats had a higher caloric intake from high fat diet during the first access than during the second access. This pattern suggests that the 2 × 1 h-HF schedule influences normal circadian rhythms and dark phase macronutrient preferences, as revealed when rats are provided with a choice of three pure macronutrients, and display a preference for carbohydrate-rich meals at dark onset, whereas during the late hours of the dark phase, protein and fat are favoured [18].

During baseline, when all rats were fed *ad libitum* on control diet, glucose and insulin concentrations increased across the light–dark transition (Fig. 5A, D), in accord with diurnal rhythms reported in *ad libitum* fed rats [19,20]. Longitudinal variation in dark phase glucose concentrations (Fig. 5B, C) appeared to reflect feeding regime rather than being secondary to change in body composition, since changes were acute in nature — there were decreases in glucose concentration across the scheduled feeding period in both schedule-fed groups. This could reflect metabolic adaptation as the rats become habituated to bingeing on the high fat diet, which is also 6.8% sucrose by energy. Studies of rats on a broadly comparable palatable feeding schedule showed no changes in glucose concentrations across a 4 h period in the middle of the light phase that encompassed a 5 g chocolate meal that was consumed in 10–15 min, suggesting that glucose was not entrainable by a palatable feeding schedule [15]. However, using the same dietary manipulation, in a separate study, blood glucose levels were shown to be elevated 30 min before a scheduled 2 h feed of 5 g chocolate [21], and may be linked to FAA. Although post-meal data were not collected, a drop in blood glucose across such a scheduled feeding period is certainly plausible.

Whereas glucose profiles appeared to be mainly influenced by scheduled feeding, insulin profiles reflected body weight gain and body fat mass, with elevated insulin concentrations in *ad libitum* HF fed rats and 2 × 1 h-HF schedule fed rats becoming apparent after 2 weeks and statistically significant after 5 weeks (Fig. 5E, F), as body weights diverge. There was no change in insulin across the scheduled feeding periods. The similarity between the HF and 2 × 1 h-HF schedule-fed groups suggests that the increased high fat diet intake in the 2 × 1 h-HF group compared to 2 h-HF may represent a tipping point towards insulin resistance. Throughout the profile studies, *ad libitum* HF fed rats were normoglycemic or hypoglycemic, and hyperinsulinemic relative to CON, which was also confirmed in the terminal blood sample (Table 1). The lower fasting glucose concentrations in rats with dietary manipulation vs. CON rats could reflect a differential effect of adaptation to HF diet on metabolic turnover or endogenous production of glucose during the 22 h fast preceding the oGTT. It is unlikely that fasting glucose levels relate to protection from insulin resistance since terminal insulin concentrations were elevated after 6 weeks on *ad libitum* HF diet (Table 1).

The development of insulin resistance suggested by the profile studies in *ad libitum* HF and 2 × 1 h-HF schedule-fed rats was substantiated by oGTT. After 4 weeks on the dietary manipulations, glucose and insulin AUCs were higher in *ad libitum* HF and both schedule-fed rat groups compared to CON (Fig. 6B, D), in line with changes in body fat mass, although there was no clear effect of the specific scheduled feeding regime. Despite the differences between the two schedule-fed groups, and the milder body phenotype compared to the HF group, the apparent loss of metabolic control, with impaired glucose tolerance and exaggerated insulin secretion, illustrates the potential of these ‘meal-fed’ models compared to the more common manipulation of obligatory feeding on a high fat diet. High fat feeding may also give rise to chronic inflammation of white adipose tissue, liver and pancreas [22], and early onset inflammation through increased cytokines may precede the development of impaired glucose handling [23]. It may be profitable to investigate these issues in the schedule-fed model. Interestingly, a scheduled feeding paradigm with restricted access to a high fat diet for 8 h during the dark phase from ZT13-21 did not lead to additional body weight gain or altered glucose metabolism. These outcomes were similar to mice fed on a low fat diet either *ad libitum* or with 8 h restricted access and contrasted with *ad libitum* high fat diet feeding [24]. This contrasts with the outcome of the current study and could reflect a species difference or a differential effect of precise feeding regime.

The gut hormones ghrelin and GLP-1 were measured in the terminal blood sample to evaluate their involvement in the anticipation of large palatable meals. Termination times of between 30 and 60 min prior to the scheduled meal in both schedule-fed groups were selected to highlight changes that might be involved in the preparation for consumption of a large meal. In the present study, we found no evidence of changes in total ghrelin or active GLP-1 concentration prior to the scheduled consumption of the palatable high fat diet (Table 1). This finding contrasts with those of studies in which a role for plasma ghrelin in the regulation of meal anticipatory processes has been suggested in rats that were trained to eat their entire daily intake from chow [17 (total ghrelin), 21 (active ghrelin), 25 (not specified)], high fat diet [21] or Ensure [25] during a short period in the mid light phase. Another gut hormone, GLP-1, has also been shown to increase prior to scheduled meals of chow [21 (active GLP-1), 26 (total GLP-1)] or high fat diet in the mid light phase [21]. However, in feeding paradigms more similar to those employed in the current study, the involvement of ghrelin and GLP-1 is less clear. Accordingly, when otherwise unrestricted rats received a chocolate snack for 15 min during the mid-light phase total ghrelin was increased at the start time of the expected snack [17], however rats receiving a chocolate meal for 2 h during the light phase, did not show an increase in active ghrelin or active GLP-1 [21].

## 5 Conclusion

We used variations on the palatable scheduled-feeding rat model first described by Berner et al. in 2008 [7], based on the dietary manipulation by Corwin et al. [27,28], to investigate temporal food intake structure, and metabolic and hormonal responses. The relevance of this model of meal-feeding with dietary choice to human obesity was supported by the increase in daily caloric intake and body fat mass on scheduled-feeding. Overall, the scheduled-feeding regimes produced a milder obesity phenotype compared to rats fed *ad libitum* on the same palatable high fat diet. Analysis of temporal feeding patterns revealed that habituation to consumption of large binge-type meals of the high fat diet did not generally reduce chow-feeding in the hours immediately prior to the scheduled feed, when increased FAA might be expected. The absence of an anticipatory hypophagic state is in accord with our previous findings that hypothalamic homeostatic systems do not appear to be involved in driving these large scheduled meals [8], raising the question of what the underlying mechanisms are, especially since the gut hormones, ghrelin and GLP-1, were also unaltered in the current study. The amount of calories consumed during scheduled access in both groups was beyond immediate homeostatic needs, which suggests that these feeding events might be reward, rather than homeostatically driven [8]. The marginal increase in overall caloric intake, and in high fat diet intake, in the 2 × 1 h-HF group resulted in greater body weight gain and elevated dark phase plasma insulin concentrations compared to the 2 h schedule-fed group, although responses to an oGTT did not differ between the groups, or between the schedule-fed groups and the HF group. Clearly, the detrimental metabolic effects of the high fat diet do not require an unremitting imposed diet or the accumulation of substantial excess body fat reserves. These findings suggest that palatable scheduled-feeding models could be useful in helping dissect both the mechanistic underpinnings of the over-consumption of calories that underlies much human obesity, and those involved in the development of metabolic disease. Further development of rodent meal-feeding models could be rewarding in both contexts.

## Figures and Tables

**Table 1:** Terminal blood hormones and metabolites after 6 weeks on dietary manipulation.

	CON	HF	2 h-HF	2 × 1 h-HF
Leptin (ng/ml)	11.91 ± 1.79^a^	32.04 ± 2.27^b^	17.86 ± 2.33^a^	17.20 ± 2.35^a^
Insulin (ng/ml)	6.91 ± 1.15^a^	11.74 ± 1.88^b^	4.78 ± 0.89^a^	5.67 ± 0.98^a^
Total ghrelin (pg/ml)	623.2 ± 89.2	505.4 ± 175.7	962.6 ± 278.6	468.2 ± 115.7
Active GLP-1 (pmol/l)	2.33 ± 0.51	2.02 ± 0.78	2.72 ± 0.65	2.53 ± 1.29
Glucose (mmol/l)	9.88 ± 0.48	9.97 ± 0.44	10.78 ± 0.62	9.74 ± 0.28
Triglyceride (mmol/l)	1.43 ± 0.12^a^	0.91 ± 0.07^b^	0.85 ± 0.12^b^	1.01 ± 0.12^b^
NEFA (mmol/l)	0.11 ± 0.02^a^	0.33 ± 0.03^b^	0.15 ± 0.02^a^	0.17 ± 0.01^a^

Data are presented as mean ± SEM. Different letters indicate *P* < 0.05 by one-way ANOVA and Student–Newman–Keul post hoc test.
